# Education and Midlife Cognitive Functioning: Evidence from the High School and Beyond Cohort

**DOI:** 10.1002/alz.088332

**Published:** 2025-01-09

**Authors:** John Robert Warren, Eric Grodsky, Chandra Muller, Jennifer J. Manly, Adam M. Brickman

**Affiliations:** ^1^ University of Minnesota, Minneapolis, MN USA; ^2^ University of Wisconsin ‐ Madison, Madison, WI USA; ^3^ University of Texas at Austin, Austin, TX USA; ^4^ Department of Neurology, Columbia University, New York, NY USA; ^5^ Columbia University, New York, NY USA

## Abstract

**Background:**

Education is associated with cognitive functioning and risk of Alzheimer’s Disease and related dementias (ADRD). However, researchers rarely consider anything about education other than highest degree completion. Do schools’ social and academic contexts and students’ other (non‐attainment) schooling outcomes independently predict cognitive outcomes? If so, this opens the possibility of manipulating educational policies and practices to improve long term cognitive well‐being.

**Method:**

This study included a nationally representative sample of 12,530 Americans followed prospectively from high school through age ∼60 as part of the High School and Beyond cohort study. OLS regression models estimated associations between various aspects of education ─ including schools’ social and academic contexts; students’ grades, test scores, and course taking; and degree attainment ─ and multiple dimensions of cognition, before and after adjustment for confounders.

**Result:**

Educational attainment predicts cognitive functioning at age ∼60. However, the social and academic contexts of schools and other measures of students’ educational performance predict cognition at least as well—even after adjusting for attainment.

**Conclusion:**

Education is more than just how far in school people go, and degree attainment is not the only ─ or perhaps even the primary ─ thing about education that has long run consequences for cognitive functioning and ADRD risk. Advancing our understanding of how and why education shapes cognitive functioning and ADRD risk requires analyses of data with nuanced information about schools, schooling, and student performance.

